# The influence of cognitive status on outcome and walking ability after hemiarthroplasty for femoral neck fracture: a prospective cohort study

**DOI:** 10.1007/s00590-016-1873-9

**Published:** 2016-10-31

**Authors:** Sebastian Mukka, Björn Knutsson, Ferid Krupic, Arkan S. Sayed-Noor

**Affiliations:** 1 Department of Surgical and Perioperative Science, Umeå University, 901 87 Umeå, Sweden; 2 Department of Orthopaedics, Institute of Clinical Sciences, University of Gothenburg, Gothenburg, Sweden

**Keywords:** Hip fracture, Femoral neck fracture, Hemiarthroplasty, Cognitive status, Outcome

## Abstract

**Introduction:**

Femoral neck fracture (FNF) is a devastating injury with serious medical and social consequences. One-third of these patients have some degree of impaired cognitive status. Despite this, a high proportion of hip fracture trials exclude patients with cognitive impairment (CI). We aimed to evaluate whether moderate to severe CI could predict walking ability, quality of life, functional outcome, reoperations and mortality in elderly patients with displaced FNF treated with hemiarthroplasty (HA).

**Methods:**

This cohort study included a consecutive series of 188 patients treated with HA for a displaced FNF. Patients were assessed for estimated preoperative and 1 year postoperatively with regard to walking ability, cognitive status, quality of life with EQ-5D and hip function with Harris hip score.

**Results:**

There were 188 patients who met the inclusion criteria. A total of 130 patients were in the control group, and 58 were in the CI group. At 1-year follow-up, 31 patients (24%) had died in the control group and 22 patients (38%) had died in the cognitive impaired group. This difference in reoperation and mortality rate was statistically significant (log-rank test, *p* = 0.016). The CI had a significantly higher incidence of being non-walker (28 vs. 4%, OR 9.2, *p* = 0.001). The EQ-5D was higher in the control group, while the Harris hip score was comparable in the two groups.

**Conclusions:**

Moderate to severe CI was associated with a high incidence of non-walking ability, worse quality of life, high mortality and re-operation rate after femoral neck fractures treated with HA.

## Introduction

Femoral neck fracture (FNF) is a devastating injury with serious medical and social consequences. Due to our aging population, the FNF incidence is high and expected to double by 2040 [1]. At least one-third of these patients have some degree of impaired cognitive status (CI) [2]. The occurrence of the fracture itself may worsen this impairment, while the presence of CI complicates the postoperative convalescence and rehabilitation. Despite this mutual negative affection, a high proportion of hip fracture trials excluded or ignored patients with CI and therefore missed an opportunity to study outcomes and identify factors associated with improved prognosis [2]. Also, patients with CI may receive less optimal treatment and rehabilitation than lucid patients although previous studies have demonstrated that mild to moderate CI does not compromise the functional gain from tailored inpatient rehabilitation during the first year after the fracture [3].

The assessment of cognitive status in FNF patients is not an easy task, especially during the acute phase when they suffer from both the fracture pain and the analgesics side effects. The assessment should therefore be undertaken using a standardized validated instrument. Parker and Palmer, for instance, assessed 882 patients with hip fractures by a new mobility score and by a mental test score, to determine which was of the most value in forecasting mortality at 1 year. Both scores gave a highly significant prediction, but the mobility score had a greater predictive value and is easier to perform [4]. Recently, this score has been further refined to determine the linear progression of functional regain and mortality prediction after hip fractures, as well as being used in research and audit studies [5]. Another commonly used test is the short portable mental status questionnaire (SPMSQ), which has been found to be useful in predicting mortality [6, 7].

In this study, we sought to evaluate whether moderate to severe CI, assessed with SPMSQ, could predict walking ability, quality of life, functional outcome, reoperations and mortality in elderly patients with a FNF treated with hemiarthroplasty (HA).

## Patients and methods

The study was conducted according to the Helsinki Declaration, and the local ethics committee approved the protocol.

In this prospective observational study, patients with a displaced FNF treated with a cemented HA Lubinus SPII^®^ (Link, Hamburg, Germany) between February 2012 and July 2014 at the Department of Orthopaedics at Sundsvall Teaching Hospital, Sweden, were considered for inclusion. Patients treated using other implants, pathological fractures, bilateral FNF fractures during the study period and hip arthroplasty for a failed internal fixation as well as bed-ridden patients were excluded. Patients were operated through either the direct lateral or the posterolateral approach.

Before the operation, patients’ baseline status was assessed for the last week before the fracture, in a retrospective rating, using the SPMSQ for the cognitive status (0–2 severe CI, 3–5 moderate CI, 6–7 mild CI and 8–10 no CI), the EQ-5D for the quality of life (−0.59 point indicates the worst possible quality of life, and 1.0 indicates the best possible quality of life) and the Harris hip score (HHS) for the hip function (0 point indicates the worst hip function, and 100 points indicate the best hip function) [8, 9]. In patients with SPMSQ score of less than 6, all clinical variables except cognitive status were assessed by means of a report from a close relative or nursing home staff as described and used by Blomfeldt et al. [10].

Data were collected regarding surgical approach, comorbidities registered at primary surgery by the American Society of Anaesthesiologists (ASA) score, early and late postoperative complications and re-operation, length of hospital stay and perioperative mortality.

An independent research nurse assessed all clinical variables at 1-year follow-up. Patients were reviewed with regard to walking ability (yes/no), quality of life with EQ-5D and hip function with HHS. The reoperation rate and 1-year mortality were identified in the hospital medical records and Swedish hip arthroplasty registry using the patient’s unique Swedish personal ID number [11].

According to the SPMSQ score, we divided the cohort into two groups. The group of patients with SPMSQ score of less than 6 was considered as moderate to severe CI group, while the group of patients with SPMSQ score of 6 or more was considered as no or mild CI. The former group will be called the CI group, while the latter group will be called the control group through the rest of the article. We compared the results of these two groups to evaluate whether moderate to severe CI could predict the ability to walk, quality of life, functional outcome and mortality.

### Statistical methods

The statistical analysis was performed with use of SPSS 22.0 for Mac software (SPSS, Chicago, IL).

Sample size was calculated based on comparing the EQ-5D of each group. With a power of 0.80 and a significance level (alpha) of 0.05, a minimum of 35 patients at follow-up were needed in each group to detect a clinically significant 40% EQ-5D reduction in the CI group.

We used Mann–Whitney *U* test to compare the ordinal and continuous variables and the Chi-square test to compare the nominal variables between the two groups. All tests were two-sided. Linear regression was used to adjust for possible confounders such as age, gender and ASA category (1–2 or 3–4) and determine factors affecting the HHS and EQ-5D. A logistic regression was performed to evaluate factors affecting walking ability, while a Cox regression analysis was performed to evaluate factors affecting mortality. The results were considered significant at *p* < 0.05.

## Results

There were 188 patients who met the inclusion criteria. According to the SPMSQ, 130 patients were in the control group and 58 were in the CI group. The patients’ baseline characteristics of the two groups are presented in Table 1 and show no differences with regard to age, sex, operated side, surgical approach, ASA class (1–2 or 3–4) or length of hospital stay. However, patients of the CI group had worse preoperative HHS and EQ-5D.

**Table 1:** Baseline characteristics of the two groups

	Control group *n* = 130	Cognitive impairment group *n* = 58	
Age	84.2 (SD 6.1)	84.9 (SD 5.6)	*p* = 0.46
Sex			
Male	36 (27.7%)	21 (36.2%)	*p* = 0.24
Female	94 (72.3%)	37 (63.8%)	
Side			
Right	65 (50.0%)	28 (48.3%)	*p* = 0.83
Left	65 (50.0%)	30 (51.7%)	
Approach			
Direct lateral	74 (56.9%)	32 (55.2%)	*p* = 0.82
Posterolateral	56 (43.1%)	26 (44.8%)	
ASA			
1–2	65 (50.0%)	24 (41.4%)	*p* = 0.27
3–4	65 (50.0%)	34 (58.6%)	
Preop HHS	83.8 (SD 11.4)	77.2 (SD 13.5)	*p* = 0.01
Preop EQ-5D	0.84 (SD 0.23)	0.62 (SD 33.5)	*p* = 0.001
Length of hospital stay (days)	13.3 (SD 8.7)	12.2 (SD 11.2)	*p* = 0.49

**Fig. 1:**
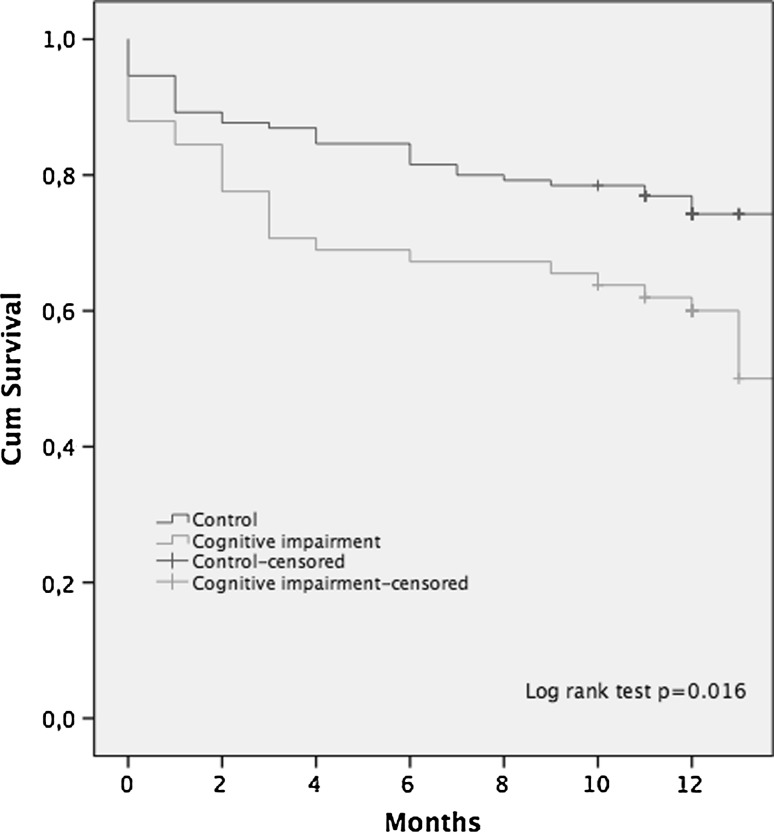
Kaplan–Meier graph comparing mortality between the groups

At 1-year follow-up, 31 patients (24%) had died in the control group leaving 99 patients in this group and 22 patients (38%) had died in the CI group leaving 36 patients in this group available. This difference in mortality was statistically significant (log-rank test, *p* = 0.016, Fig. 1). The adjusted Cox regression analysis showed that there was an increased mortality (with tendency to be statistically significant) during the study period in the CI group [HR 1.66 (95% CI 0.99–2.81), *p* = 0.06]. Higher age was also associated with a higher mortality [HR 1.08 (95% CI 1.03–1.13), *p* = 0.001] (Table 2).

**Table 2:** Mortality at 1-year follow-up

Variable	HR	2.5–97.5%
Cognitive status		
Control	1.00	Ref
Cognitive impairment	1.66	0.99–2.81, *p* = 0.06
Age	1.08	1.03–1.13, ***p*** **=** **0.001**
Sex		
Male	1.00	Ref
Female	0.81	0.47–1.40, *p* = 0.46
ASA		
1–2	1.00	Ref
3–4	1.04	0.62–1.75, *p* = 0.89

Cox proportional hazard including adjusted variables and presented as hazard ratio

The bold value is significant at *p* < 0.05

Regarding walking ability, the CI group had a significantly higher incidence of being non-walker [28 vs. 4%, OR 9.2 (95% CI 2.63–32.7), *p* = 0.001]. The logistic regression analysis showed the CI status to be the only factor that results in becoming non-walker (Table 3). The EQ-5D was higher in the control group (control group vs. cognitive impairment group, 0.70 vs. 0.46, *p* = 0.001), while the HHS was comparable in the two groups (control group vs. cognitive impairment group, 73 vs. 67, *p* = 0.135). The regression analysis showed the control group cognitive status and preoperative HHS and EQ-5D to be the only factors that improved EQ-5D (Table 4).

**Table 3:** Risk for postoperative non-walker

Variable	Non-walker	
	OR	2.5–97.5%
Surgical approach		
Posterolateral	1.00	Ref
Direct lateral	1.37	0.40–4.74, *p* = 0.61
Cognitive status		
Control group	1.00	Ref
Cognitive impairment	9.20	2.63–32.17, ***p*** **=** **0.001**
Age	0.99	0.90–1.10, *p* = 0.89
Sex		
Male	1.00	Ref
Female	1.05	0.28–3.95, *p* = 0.94
ASA		
1–2	1.00	Ref
3–4	1.12	0.34–3.72, *p* = 0.86

Logistic regression presenting adjusted odds ratio

The bold value is significant at *p* < 0.05

**Table 4:** Outcome variables

Variable	EQ-5D		HHS	
	OR	2.5–97.5%	OR	2.5–97.5%
Surgical approach				
PL approach	1.00	Ref	1.00	Ref
DL approach	−0.03	−0.15 to 0.10, *p* = 0.65	−0.40	−6.3 to 5.7, *p* = 0.92
Cognitive status				
Cognitive impairment	1.00	Ref	1.00	Ref
Control group	0.22	0.08–0.35, ***p*** **=** **0.002**	4.65	−2.24 to 11.53, *p* = 0.18
Age	−0.02	−0.02 to 0.001, *p* = 0.06	−0.21	−0.74 to 0.33, *p* = 0.44
Sex				
Male	1.00	Ref	1.00	Ref
Female	0.06	−0.07 to 0.19, *p* = 0.43	1.02	−4.9 to 8.5, *p* = 0.59
ASA				
1–2	1.00	Ref	0.00	Ref
3–4	−0.07	−0.19 to 0.05, *p* = 0.26	0.07	−6.2 to 6.4, *p* = 0.98
Preop EQ-5D/HHS	0.16	−0.69 to 0.39, *p* = 0.17	0.34	0.08 to 0.6, ***p*** **=** **0.01**

Linear regression including adjusted variables for HHS and EQ-5D

The bold value is significant at *p* < 0.05

We also found a significant difference in reoperation rate between the groups in favor of the control group (Table 5).

**Table 5:** Re-operation in the two groups

	Control group (*n* = 130)	Cognitive impairment (*n* = 58)
Excision arthroplasty due to dislocation	0	1
THA with dual mobility cup due to dislocation	4	1
Surgical debridement due to deep infection	3	6
Excision arthroplasty due to deep infection	0	2
Open reduction and internal fixation of periprosthetic fracture	1	0
Secondary total hip arthroplasty due to acetabular erosion	1	0
Number of hips with re-operation^a^	9 (6.9%)	10 (13.8%)

^a^Adjusted OR 3.16 (95% CI 1.17–8.55), *p* = 0.02

## Discussion

This study showed that moderate to severe CI, compared to no or mild CI, was associated with higher risk of becoming non-walker, worse quality of life, higher mortality and higher re-operation rate after FNF treated with HA. However, the overall 1-year hip function in both groups was comparable.

There is paucity in the literature regarding the evaluation of FNF outcome in CI patients. In a systematic review, Mundi et al. [2] showed that these patients were seldom included (26%) and rarely the focus (1%) of RCTs evaluating operative FNF management. Only 3% of the included studies had reported outcomes specific to CI patients. Their conclusion criticized the external validity of the existing evidence and called for inclusion of patients with CI to identify interventions that improve survival and function in this patient population.

The most suitable treatment option for displaced FNF in patients with CI has been a matter of debate. Some clinicians consider this group of patients as a high-risk group with low functional demand, therefore recommend fracture reduction and screw fixation for instance and found that HA in demented patients was a too major operation and less invasive methods of internal fixation should be considered [12]. Others have reported better postoperative walking ability and functional outcome with HA, even in the presence of severe CI [13–16]. As per our department’s guidelines, we treated all patients with displaced FNF, regardless of cognitive status, with hip arthroplasty as far as no medical contra-indication existed.

We chose the SPMSQ score to determine the cognitive status. This score was previously found quick, easy to administer and reliable. It has also been validated as having a similar sensitivity and specificity to the Mini-Mental State Examination and as a severity-rating instrument [17–19].

We think the cutoff value of <6 (moderate to severe CI) and ≥6 (no to mild CI) was a suitable one since it differentiated between the group of lucid patients and patients with mild CI caused by mild dementia or secondary to the influence of the fracture and its management with analgesics and hospital admission and the group of patients with more prominent CI most likely caused by moderate to severe dementia [20].

Our results showed similar baseline characteristics of the control and CI groups. However, patients of the CI group had worse preoperative HHS and EQ-5D (Table 1). The latter finding concurs with those reported by Söderqvist et al. [21]. Postoperatively, the mortality was significantly higher in the CI group at 1-year follow-up. When adjusting for possible predictive factors, older age was significantly associated with a higher mortality. CI showed a tendency to give a higher mortality, although this did not reach a statistical significance [HR 1.66 (95% CI 0.99–2.81), *p* = 0.06] (Table 2). These results are in agreement with those reported by others including those who used SPMSQ score <3 as a cutoff value, i.e., compared patients with severe CI with others [21, 22].

The quality of life at 1-year follow-up, as evaluated by EQ-5D, showed better results in the control group. No other factor influenced the EQ-5D, apart from age, which only showed a tendency for influence [OR −0.02 (95% CI −0.02 to 0.001), *p* = 0.07]. Parsons et al. [23] found that EQ-5D could be used to measure outcome for patients recovering from hip fracture, including those with CI. The same group of researchers reported that there was strong evidence that quality of life was lower for patients with CI [24]. Söderqvist et al. [20] made the same observation founding a lower EQ-5D in patients with severe CI group both preoperatively and 1-year postoperatively. This difference in quality of life is probably related to the general physical and mental status of patients of the two groups.

The present study showed no statistically significant difference in the functional outcome, as evaluated by HHS, between the two groups at 1-year follow-up. On the other hand, CI was the only predictive factor that associated being non-walker at 1-year follow-up. More than one out of four patients alive at the 1-year follow-up with CI were a non-walker. The comparative number of the lucid patients was 4%. This deterioration in walking ability was also found by other studies. Muir et al. [25], for instance, reported in their systematic review that the presence of CI adversely affected walking ability and function, mainly in patients treated surgically with internal fixation after femoral neck fracture and not (or to a much lesser extent) in patients treated with HA. They recommended intensive inpatient rehabilitation for these patients to reach comparable gains as with lucid patients. This is important not just for the individual but also for the society as a wheelchair or bedridden patient demands a higher level of assistance on the daily basis.

We determined a higher re-operation risk in the CI group mainly due to surgical debridement of deep infection [adjusted OR 3.16 (95% CI 1.17–8.55) *p* = 0.02]. Similarly, Strömberg et al. [20] found that CI was associated with an increased complication rate, e.g., a three-fold increase of early FNF displacement and a fourfold increase of wound infection. This increased risk was present even in patients with mild to moderate CI and could not entirely be explained by age. The increased rate of infection and dislocation further contributes to the higher mortality in the CI group [26]. Furthermore, Mariconda et al. [27] found that comorbidities and poor cognitive status could determine the likelihood of early and delayed general complications, respectively. In contrast to these observations, Lapidus et al. [28] found that age, gender, cognitive function, ASA classification, or the time to surgery had no influence on reoperation risk due to fracture healing complication.

The present study has some limitations. The 1-year follow-up time is relatively short. We think that the short-term follow-up is the most important in FNF patients owing to their high complication rate and mortality during the first postoperative year. The used HHS and EQ-5D scores have some disadvantages, e.g., the ceiling effect that could mask some of the differences among patients. Finally, we did not evaluate the effect of rehabilitation on the outcome in different groups and therefore cannot approximate if such an effect existed. These limitations are compensated by the strengths of the study, which is a prospective cohort study with consecutive patients, adequate sample size, minimal dropout and enlightening an important and increasing group of patients, which often are excluded from research studies. An independent nurse conducted the follow-up in all patients, minimizing potential bias. These factors improve the generalizability of the results.

In conclusion, moderate to severe CI, compared to no or mild CI, was associated with a high incidence of non-walking abilities, worse quality of life, higher mortality and a higher re-operation rate after FNF treated with HA, compared to the patients with mild or no cognitive impairment. Future studies could focus on how we can improve the walking abilities and decrease the mortality among patients with cognitive impairment after arthroplasty for a displaced FNF.
